# Influence of Sucrose and Immersion Time on *Humulus lupulus* L., cv Columbus, Plantlet *In Vitro* Proliferation and Potentially Bioactive Compound Content

**DOI:** 10.3390/plants14040537

**Published:** 2025-02-10

**Authors:** Valeria Gianguzzi, Leandra Leto, Anna Agosti, Andrea Di Fazio, Francesco Paolo Marra, Martina Cirlini, Benedetta Chiancone

**Affiliations:** 1 Department of Agricultural, Food and Forest Sciences (SAAF), University of Palermo, 90128 Palermo, Italy; valeria.gianguzzi@unipa.it (V.G.); francescopaolo.marra@unipa.it (F.P.M.); 2 Department of Food and Drug, University of Parma, Viale Parco Area delle Scienze 27/A, 43124 Parma, Italy; leandra.leto@unipr.it (L.L.); anna.agosti@unipr.it (A.A.); andrea.difazio@studenti.unipr.it (A.D.F.); benedetta.chiancone@unipr.it (B.C.); 3 Institute of Biophysics, National Research Council (CNR), Via Ugo La Malfa 153, 90146 Palermo, Italy

**Keywords:** hop (*Humulus lupulus* L.), micropropagation, Plantform^TM^ bioreactor, temporary immersion system (TIS)

## Abstract

(1) Background: Traditionally, hop is propagated using rhizome fragments or herbaceous stem cuttings. Micropropagation, therefore, offers a viable alternative for the large-scale production of healthy, genetically uniform plants, regardless of the season and within confined spaces. A temporary immersion system (TIS) facilitates plant propagation by alternating immersions of microcuttings in liquid culture medium with dry periods, preventing gas accumulation through forced ventilation. (2) Methods: In this study, the response of hop plantlets, cv. Columbus, cultured in media with and without sucrose, in solid and liquid culture systems (Plantform^TM^ bioreactor), was evaluated, considering its effect on morpho-physiological parameters, on the total phenolic content, and on antioxidant capacity. Moreover, to make the TIS more efficient, the effect of immersion duration (three and six minutes every twelve hours) was evaluated. (3) Results: The presence of sucrose in the culture medium improved plant proliferation in both culture systems tested: solid and liquid (particularly for explants immersed for three minutes). In the TIS, plantlets with a higher antioxidant capacity were obtained when sucrose-free culture medium was used. (4) Conclusions: This study confirms the efficacy of the TIS as a hop propagation method but also as a valid tool to produce biomass to be used as a source of bioactive compounds.

## 1. Introduction

Hop (*Humulus lupulus* L.) is a species belonging to the *Cannabaceae* family [1,2]. Only female plants are used for the cultivation of hops as they produce inflorescences, called cones, which are a fundamental ingredient for the production of beer. The secondary metabolites produced by hop plants (mainly flavonoids, bitter acids, and essential oils) show not only a benefit for beer production but also have great value for both the food and pharmaceutical industries [3,4,5]. These sectors are increasingly exploring the potential of plant-based additives due to the diverse and remarkable bioactivities they offer, such as antioxidant and antimicrobial properties, among others [6,7]. Hop cultivation is widespread throughout the world; in Italy, the surface area dedicated to hops is limited (60 ha) [8]. The traditional propagation of hop plants and the use of non-certified plants are some of the factors limiting the diffusion of this species in Italy. Traditionally, hop is agamically propagated using fragments of rhizome or herbaceous stem cuttings that, during the autumn or spring months, are planted in open fields, directly or after a brief period in pots [9]. However, these methods cannot meet the growing demand for plant material from farmers because there is limited availability of suitable rhizomes, which hinders the adoption of this method [10,11]. Therefore, micropropagation can be a valid alternative for the large-scale propagation of healthy and genetically uniform plants, regardless of the season and in limited space. Recent studies have demonstrated that *in vitro* hop plantlets synthetize the same bioactive compounds present in those grown in open fields [10,11]. *In vitro* tissue culture techniques offer the possibility of controlling the explant growing conditions; it has been demonstrated that the methods used for culturing plants can act as triggers for the secondary metabolism, thereby boosting the production of bioactive compounds [12].

Although some previous research has been conducted for hop micropropagation [13,14,15,16], considerable efforts are still needed to improve micropropagation protocols, reducing the time needed to multiply the material and improving its proliferation. To define a micropropagation protocol, several aspects come into play from the selection of the mother plants to the disinfection of explants: the induction, multiplication, growth, rooting, and adaptation to *ex vitro* conditions [17]. Each of these steps must be studied to ensure the efficiency of the propagation protocol. One of the most important stages of the micropropagation process is the multiplication phase, which determines the efficiency and cost of production plants. This phase is usually performed in solid culture medium. However, the use of gelling agents, together with the manual labor for the numerous subcultures needed, makes the micropropagation process more expensive. A valid alternative is resorting to a liquid culture medium system that can provide much more uniform culturing conditions; moreover, the media can be renewed without changing the container, sterilization is possible by microfiltration, and it can be completely automatized [18].

In recent years, to perform micropropagation in liquid culture, temporary immersion systems (TISs) have been considered more effective [19,20,21,22,23,24]; moreover, thanks to the temporary and short-term contact of the explant with the liquid culture medium, TISs provide an adequate supply of oxygen to plants in culture [18] and reduce the problem of hyperhydricity [20]. Hyperhydricity refers to the problem of the accumulation of excess water in plants, meaning that they take on a glassy appearance due to reduced chlorophyll content and the anomalous development of chloroplasts [25,26].

The TIS has been used for the micropropagation of several species such as *Stevia rebaudiana* [27], *Colocasia esculenta* L. Schott [28], *Agave angustifolia* [29], *Rosmarinus officinalis* L. [30], *Dracocephalum forrestii* [31], *Guarianthe skinneri* [32], *Cannabis sativa* L. [33], and also for hop [34].

To be able to optimize an efficient propagation technique using the TIS, it is necessary to take into account various cultural parameters such as the frequency and duration of immersion, the volume of the liquid medium, the volume of the culture container, the number of explants, ventilation, and forced ventilation [18].

To perform micropropagation in the TIS, several bioreactor types can be used; among them, the most recent is Plantform^TM^. These bioreactors are easy to use, autoclavable, transparent, and easy to carry [35,36].

*In vitro* plant cultures are usually not autotrophic as they need carbohydrates as a source of carbon and energy. The most applied is sucrose that can act as a signaling molecule and maintain osmotic potential in cells, but at high concentrations, may decrease net photosynthesis and can cause abnormalities [37]. It has been reported that the photosynthetic capacity of tissues is improved when they are grown in bioreactors with forced ventilation compared to other *in vitro* culture methods [38], so it is possible to have the opportunity to reduce or even eliminate the traditional integration of sugar, producing photoautotroph cultures [37]. Therefore, in this study, in order to set up an efficient micropropagation method for hop, the response of plantlets of cv. Columbus, a popular American hop variety with a high acid content, cultured in media with and without sucrose, in solid and liquid culture systems (Plantform^TM^ bioreactor), was studied. Moreover, to make the TIS a valid alternative to culture on solid medium, the effect of immersion duration (three minutes and six minutes) was evaluated in relation to the morpho-physiological and biochemical parameters of the plantlets.

## 2. Results

### 2.1. Experiment 1: Effect of Sucrose Supplementation on Morpho-Physiological and Biochemical Parameters of Hop Plantlets Grown on Solid Culture Medium

**Figure 1:**
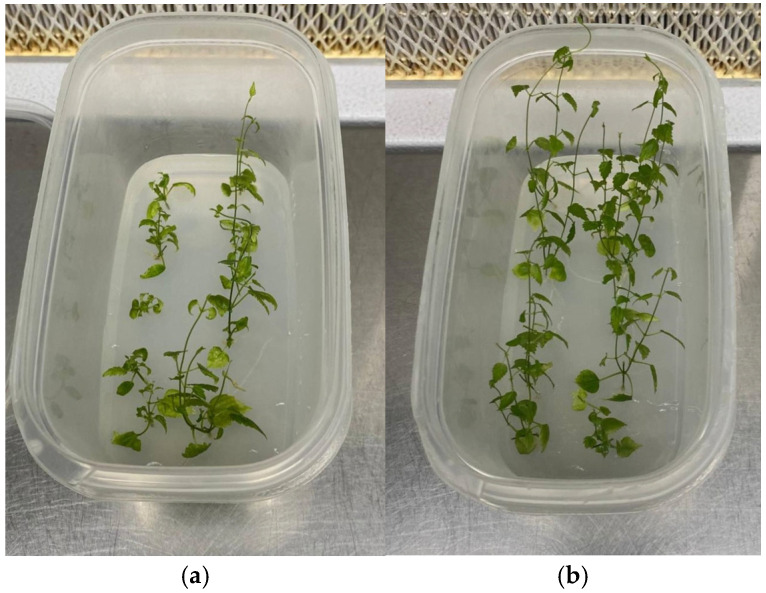
*In vitro* propagation of *Humulus lupulus* L., cv Columbus, after 4 weeks of culture. (**a**) Explants grown in the sucrose-free medium (PMSF) and (**b**) explants grown in the medium with sucrose (PM).

In this experiment, the influence of sucrose in the culture medium was studied. During the experiment, the explants remained viable, independently of the presence of sucrose in the medium, maintaining a green color for the entire culture. After 12 days of culture, the explants started to develop shoots and, at the same time, roots. The analysis of the data recorded to evaluate the proliferation of hop plantlets and their physiological and biochemical response grown in solid medium in the presence and absence of sucrose (Figure 1) is reported in Table 1 and Table 2.

The obtained results showed that the culture medium’s composition did not significantly influence any of the following parameters: number and length of shoots, number of roots, relative growth rate (RGR), index of chlorophyll content in leaf (SPAD), total (poly)phenolic content (TPC), and antioxidant capacity (Table 1 and Table 2); instead, a significant influence was registered on the parameters “sprouting”, “rooting”, and “length of roots” (Table 1). Specifically, in the medium containing sucrose, statistically higher percentages of sprouting and rooting and the longest roots were recorded (Table 1).

### 2.2. Experiment 2: Effect of Sucrose Supplementation and Immersion Time on Morpho-Physiological and Biochemical Parameters of Hop Plantlets Grown on TIS

**Figure 2:**
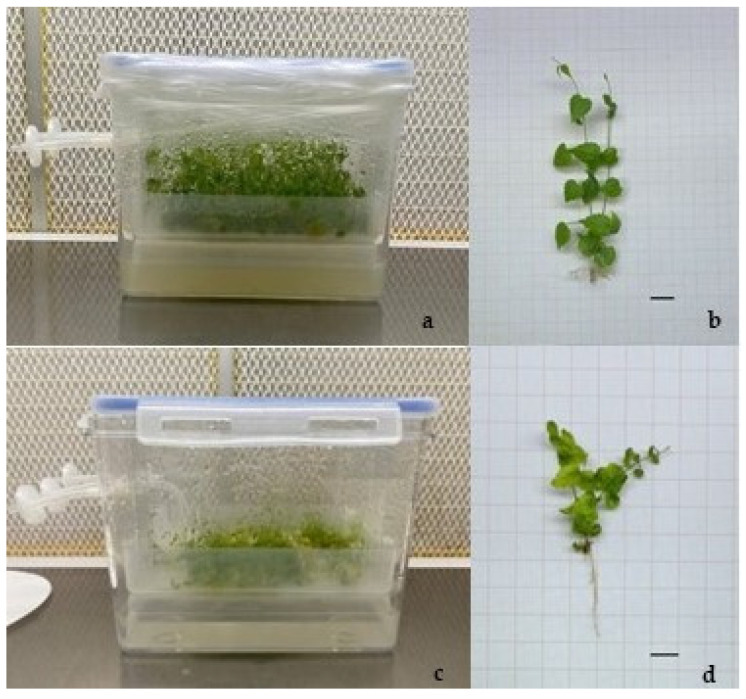
Explants of hop, cv Columbus, cultured in the Plantform^TM^ bioreactors after four weeks of culture, with (**a**,**b**) 3 min/12 h and (**c**,**d**) 6 min/12 h of plantlet immersion in the culture medium, enriched with sucrose. Bars = 1 cm.

After 6 days in culture, the explants placed in the Plantform^TM^ bioreactor started to develop shoot and roots, maintaining a green color for the entire time of culture (Figure 2).

Table 3 shows the effect of sucrose supplementation and the immersion time on the *in vitro* proliferation of hop plantlets and on their physiological and biochemical parameters.

The statistical analysis evidenced that the two considered factors influenced the parameters “sprouting”, “number of shoots”, “rooting”, “number of roots”, and “length of shoots” independently of each other (Table 3). For this reason, per these parameters, the following values are the averages calculated within the single factor. Specifically, considering “culture medium composition”, explants cultured on sucrose-enriched medium showed a better response than those grown in sucrose-free medium; in more detail, there was the highest average of sprouting (86% ± 0.04 vs. 61% ± 0.06) with the development of the number and length of shoots (on average 1.78 ± 0.06 vs. 1.33 ± 0.07; 4.11 ± 0.20 cm vs. 2.08 ± 0.27 cm, respectively). Moreover, in culture media enriched with sucrose, the highest average of rooting (85% ± 0.04 vs. 53% ± 0.06) and mean of the number of roots (3.81 ± 0.23 vs. 2.54 ± 0.31) were observed.

Furthermore, it was observed that, on average, the percentages of explant sprouting (85% ± 0.04 vs. 63% ± 0.06) and rooting (85% ± 0.04 vs. 53% ± 0.06), as well as the mean number of shoots per explant (1.68 ± 0.06 vs. 1.44 ± 0.07), the mean number of roots per explant (4.17 ± 0.23 vs. 2.18 ± 0.31), and the mean shoot length (4.34 ± 0.21 cm vs. 1.85 ± 0.26 cm), were statistically higher for the 3 min/12 h immersion time rather than those observed at 6 min/12 h.

For the parameters “length of roots”, a statistically significant interaction was observed between the two factors studied. Specifically, when sucrose was added to the culture medium, the immersion time did not influence the length of the roots, while in sucrose-free medium, explants immersed for 3 min/12 h produced roots statistically longer than those immersed for 6 min/12 h (1.44 ± 0.11 cm vs. 0.54 ± 0.27 cm, respectively). Moreover, for both immersion times considered, the presence of sucrose in the cultured medium induced the production of roots statistically longer than in sucrose-free medium (Table 3).

The statistical analysis evidenced a significant interaction between the factors also for the parameter RGR (Table 3). In detail, in the sucrose-added culture medium, the RGR recorded was significantly higher than that in the sucrose-free medium (21.33 mg g^−1^ d^−1^ ± 0.24 vs. 3.96 mg g^−1^ d^−1^ ± 0.47 ), but only if explants were immersed for 3 min/12 h; when the time of immersion increased to 6 min/12 h, no more differences were observed (7.19 mg g^−1^ d^−1^ ± 0.43 vs. 4.36 mg g^−1^ d^−1^ ± 0.54) (Table 3). Comparing the results within the explants grown in sucrose-enriched medium, the RGR of the explants immersed for 3 min was 3-fold higher than that of those immersed for 6 min (21.33 mg g^−1^ d^−1^ ± 0.24 vs. 7.19 mg g^−1^ d^−1^ ± 0.43) (Table 3).

Immersion time was the only factor influencing the SPAD index (Table 3), with values statistically higher recorded in explants immersed in the culture medium for 3 min rather than for 6 min (21.94 ± 1.12 vs. 18.14 ± 1.14 on average, respectively).

Immersion time was the only factor that also influenced the TPC parameter (Table 4); explants immersed for 3 min/12 h synthesized (poly)phenols in a statistically higher amount than those immersed for 6 min/12 h (on the average 5.10 mg GAE/g ± 0.09 vs. 4.65 mg GAE/g ± 0.12) independently of the presence of sucrose in the culture medium.

Finally, a significant interaction was observed for the parameter antioxidant capacity (AO) (Table 4); when sucrose was added to the culture medium, the immersion time did not influence the antioxidant capacity of the explants (5.55 ± 0.23 vs. 5.60 ± 0.23). Instead, in the absence of sucrose, the shorter immersion time determined an antioxidant capacity 2-fold higher than the longer one (4.97 mg TEAC/mL ± 0.23 vs. 2.31 mg TEAC/mL ± 0.33); furthermore, only for explants immersed for 6 min in sucrose-free culture medium was the lowest antioxidant value observed (2.31 mg TEAC/mL ± 0.33) (Table 4).

## 3. Discussion

The aim of this study was to investigate the morpho-physiological and biochemical response of hop plantlets grown in solid and liquid media (TIS) in the presence or absence of sucrose; moreover, in the TIS, the influence of immersion time was evaluated.

The results of this study confirm what was reported for other species [22,23,39,40], namely that a better plantlet response is obtained in the TIS rather than in the solid medium system. In this study, the explants grown in the TIS started to sprout and root in 6 days, instead of in 12 days, as observed in solid medium. The improved performance of plants cultured in bioreactors is attributed to the increased gas exchange and oxygen availability due to the forced ventilation induced [24,35] that facilitates the photosynthetic ability of tissue; this outcome can lead to a reduction in or the elimination of sucrose in culture media, with a consequent reduction in micropropagated plants [37,38].

The composition of the culture medium plays an important role in plant growth in both the solid and liquid systems. In some cases, the liquid culture present in the TIS can contribute to the use of lower mineral salts and PGRs compared to the conventional culture in solid medium. In fact, the use of liquid media, other than reducing the production costs by eliminating the gelling agent [41], contributes to a more efficient use of the culture medium components since direct contact with the medium makes nutrient absorption more efficient for explants [38,42,43]. Normally, sucrose is necessary for plant growth, as a signaling molecule within the plant metabolism, among other physiological processes [44]. For most plant tissue culture protocols, sucrose is the carbon source used as it is an easily assimilated macronutrient that quickly provides energy [45].

In the present study, the morpho-physiological and biochemical response of hop plantlets was influenced by the presence of sucrose, both in the solid and liquid culture systems. In the solid system, even if the presence of sucrose determined a higher sprouting (%) and rooting (%) and length of shoots compared to the medium without sucrose, it must be highlighted that the parameters analyzed were not significantly influenced by the presence of sucrose. This can be considered a promising achievement in the path of reducing hop micropropagation costs.

Conversely, in the TIS, the presence of sucrose in the media is crucial for plant proliferation; in fact, higher sprouting (%) and rooting (%) with the development of the highest number of shoots and roots and the length of shoots were observed in plants grown in media enriched with sucrose. Similar results were obtained in *Vernonia condensata* [46] and *Fraxinus mandshurica* [47], for which the number of shoots increased when the medium was supplemented with sucrose. Different results were obtained in *Bambusa vulgaris* [48], *Paulownia fortunei* [49], and *Salix viminalis* L. [40], where the number of shoots increased when a sucrose-free medium was used.

In every system of TIS liquid culture, the selected cycle of immersion is the most critical parameter influencing system efficiency. The current results showed a greater proliferation when explants were immersed for 3 min instead of 6 min every 12 h. This result could, perhaps, be caused due to an increase in physiological disorders, including asphyxia and hyperhydricity, due to the longer immersion time [50].

The influence of immersion period duration has been studied on several species, influencing the proliferation rate of the explants, such as in *Hypoxis argentea* L. when the explants were immersed for 6 min/12 h [51] in the Plantform^TM^ bioreactor; in *Olea europaea* L., the olive shoots propagated in Plantform™, with an immersion frequency of 8 min every 16 h and additional ventilation, showed high growth rates [21]; and in *Myrtus communis* (L.), a multiplication rate of 11.40 was obtained in the Plantform^TM^ bioreactor with a duration of immersion of 15 min/8 h [52]. The shoot multiplication rate of *Coffea* was also influenced by the immersion times tested; in fact, increasing the immersion time from 1 to 5 min every 6 h registered an increase of 1.5 in the plant multiplication rate [53].

Several authors have confirmed that the temporary immersion system is an effective tool to obtain secondary metabolites [35]. In this work, the parameter TPC was influenced only by immersion duration. The higher value of TPC was obtained when the explants were immersed for 3 min/12 h rather than for 6 min/12 h, independently of the presence of sucrose in the culture medium. The influence of immersion time on TPC has also been found for other species such as *Castilleja tenuiflora* [54] and *Lycium barbarum* (L.) [55], in which the (poly)phenol content was higher or lower depending on the genotype and the duration of immersion. The parameter AO was not influenced by immersion time; instead, in the absence of sucrose, the shorter immersion time determined an antioxidant capacity 2-fold higher than the longer one. This difference in the results could maybe be due to the genotype-dependent capacity of nutrient absorption [55] and secondary metabolism activation. The high quality of the plants obtained and the high growth rate compensate for the differences in the physiological and biochemical parameters.

## 4. Materials and Methods

### 4.1. Plant Material

Plantlets of hop, cv Columbus, grown *in vitro*, were used for this study.

The explants, uninodal microcuttings, were isolated from plants grown in 500 mL glass jars on proliferation medium (PM), the composition of which is as follows: MS medium 1× [56], with 30 g L^−1^ sucrose and 8 g L^−1^ plant agar (Duchefa, Haarlem, The Netherlands). The pH of the media was adjusted to 5.8.

### 4.2. Experimental Design

This research consisted of two experiments investigating the effects of different cultural conditions on the morpho-physiological and biochemical parameters of hop plantlets grown in solid and liquid culture media.

Experiment 1: The effect of sucrose supplementation on the morpho-physiological and biochemical parameters of hop plantlets grown on solid culture medium.

**Figure 3:**
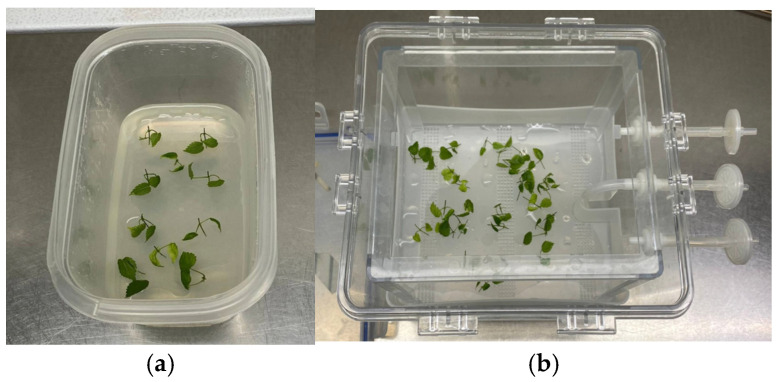
Culture of hop, cv Columbus, uninodal microcuttings (**a**) in solid medium and (**b**) in Plantform^TM^ bioreactors.

In order to evaluate the influence of sucrose in the culture medium, uninodal microcuttings were cultured on sucrose-free medium (PMSF) and with sucrose (PM). Per each type of culture media, five Microbox ECO_2_ containers (Combiness, Belgium-Micropoli, Italy) with 10 explants each were disposed (See Figure 3). The cultures were placed in a climate growth chamber at 25 ± 1 °C under a photoperiod of 16 h per day with a light intensity of 50 µmol m^−2^ s^−1^.

Experiment 2: The effect of sucrose supplementation and immersion time on the morpho-physiological and biochemical parameters of hop plantlets grown on the TIS. 

To carry out this experiment, previously sterilized TIS Plantform^TM^ bioreactors [57], containing 500 mL of culture medium, were used [36]. Per each type of culture media, two Plantform^TM^ bioreactors, with 25 explants each, were disposed. The system was set for one air change per day for five minutes; air injection was performed using 0.20 μ pore filters to promote sterilization [18]. The cultures were placed in a climate growth chamber at 25 ± 1 °C under a photoperiod of 16 h per day with a light intensity of 50 µmol m^−2^ s^−1^.

The influence of sucrose in the culture medium and the duration of immersion was studied considering the following treatments: (1) PM without agar (LPM) and 3 min/12 h of plantlet immersion; (2) LPM and 6 min/12 h of plantlet immersion; (3) LPMSF without sucrose and 3 min/12 h of immersion; and (4) LPMSF without sucrose and 6 min/12 h of immersion. 

### 4.3. Data Collection

Per each experiment, after 4 weeks of culture, the following parameters were recorded: sprouting (%) (n° of explants with shoots/n° of explants in culture × 100), number and length of shoots, rooting (%) (n° of explants with roots/n° of explants in culture × 100), number and length of roots, RGR, and SPAD (measured with Multi-Pigment Meter, MPM-100S, Opti-Sciences, Hudson, NH, USA).

### 4.4. Evaluation of Total Phenolic Content and Antioxidant Capacity

#### 4.4.1. Sample Extraction

Before the extraction step, the four-week-old hop plantlets were washed with distilled water to remove any residues of agar and then weighed and freeze-dried with a Lio-5P lyophilizer (5Pascal, Milan, Italy). The lyophilized material was then powdered using a pestle and mortar. The resulting powder (0.5 g) was suspended in 10 mL of an 80/20 ethanol/water solution (*v*/*v*). The extraction was conducted as reported by Leto et al. [11] and the supernatants were diluted (1/5 ratio with 80/20 ethanol/water mixture) before the spectrophotometric tests [10]. All the results reported are expressed on dry matter (DM).

#### 4.4.2. Total Phenolic Content and Antioxidant Capacity Determination

The determination of the TPC and AO of the sample extracts was carried out using different spectrophotometric tests. With regard to the determination of the polyphenol content, the Folin–Ciocalteu reagent-based test was applied, while the AO determination was achieved by the application of the 2,2-Diphenyl-1-picrylhydrazyl (DPPH) assay, following the indications of Leto et al. [11]. All the analytical parameters concerning the calibration of the method, the reaction steps with the reagent, and the instrumental measurements are the same as those described by Chiancone et al. [10]. All data were calculated on dry matter (DM), and measurements were performed using a JASCO V-530 spectrophotometer (Easton, MD, USA), with characteristic absorbance values set for each test and all samples measured in triplicate.

### 4.5. Statistical Analysis

For experiment 1, Student’s *t*-test (*p* < 0.05 level) was performed to determine the influence of the presence of sucrose in the solid culture medium on the different parameters evaluated.

In the second experiment, a two-way analysis of variance (ANOVA), at the *p* ≤ 0.05 level, was performed to determine the influence of the factors “culture medium composition” and “immersion time” on the parameters recorded. Statistical analysis was carried out using SYSTAT 13 software, and mean separation was carried out by Tukey’s test (*p* < 0.05 level).

## 5. Conclusions

According to the results obtained in this study, the use of the Plantform™ bioreactor improved the *in vitro* proliferation of hop, cv Columbus. The addition of sucrose in the culture medium has been confirmed as mandatory for improving plant proliferation in the TIS but not in the solid medium system. Moreover, a higher total (poly)phenol content was observed in the plantlets grown in the TIS than in those grown in solid medium, especially in the presence of sucrose. In fact, a 6 min immersion time was found to be detrimental to (poly)phenol accumulation and antioxidant capacity.

This study forms the basis for further investigations into the possibility of reducing or even eliminating sucrose in the culture medium in order to reduce the cost of micropropagation. In addition, it would be worthwhile investigating the composition of the culture medium and the frequency and duration of immersion in the TIS in order to increase the multiplication rate and the synthesis of secondary metabolites.

## Figures and Tables

**Table 1:** Effect of culture medium on the morpho-physiological parameters of *Humulus lupulus* L., cv Columbus, on plantlets grown in solid medium (mean + standard error) after 4 weeks of culture.

Culture Medium Composition	Sprouting (%)	Number of Shoots (n°)	Length of Shoots (cm)	Rooting (%)	Number of Roots (n°)	Length of Roots (cm)	RGR (mg g^−1^d^−1^)	SPAD
PM	100 ± 0.00 a	1.60 ± 0.11 a	4.09 ± 0.27 a	96 ± 0.03 a	4.06 ± 0.31 a	1.22 ± 0.06 a	7.69 ± 1.09 a	23.53 ± 0.96 a
PMSF	80 ± 0.13 b	1.33 ± 0.09 a	3.31 ± 0.46 a	56 ± 0.09 b	3.58 ± 0.31 a	0.95 ± 0.05 b	11.03 ± 3.94 a	22.14 ±1.31 a

Within each column, values followed by different letters are statistically different according to Student’s *t*-test (*p* < 0.05). PM: proliferation medium; PMSF: proliferation medium without sucrose. RGR: relative growth rate; SPAD: index of chlorophyll content in leaf.

**Table 2:** Effect of culture medium on the biochemical parameters of *Humulus lupulus* L., cv Columbus, on plantlets grown in solid medium (mean + standard error) after 4 weeks of culture.

Culture Medium Composition	TPC (mg GAE/g)	AO (mg TEAC/g)
PM	4.13 ± 0.07 a	4.00 ± 0.20 a
PMSF	4.49 ± 0.14 a	4.08 ± 0.30 a

Within each column, values followed by different letters are statistically different according to Student’s *t*-test (*p* < 0.05). PM: proliferation medium; PMSF: proliferation medium without sucrose. TPC: total (poly)phenolic content; AO: antioxidant capacity.

**Table 3:** Effect of culture medium on the morpho-physiological parameters of *Humulus lupulus* L., cv Columbus, plantlets grown in temporary immersion system (mean + standard error) after 4 weeks of culture.

Immersion Time	Culture Medium Composition	Sprouting (%)	Number of Shoots (n°)	Length of Shoots (cm)	Rooting (%)	Number of Roots (n°)	Length of Roots (cm)	RGR (mg g^−1^d^−1^)	SPAD
3 min/12 h	LPM	100 ± 0.00	1.93 ± 0.08	5.28 ± 0.25	100	4.53 ± 0.30	1.83 ± 0.09	21.33 ± 0.27	22.14 ± 1.62
	LPMSF	70 ± 0.08	1.42 ± 0.10	3.40 ± 0.35	70	3.81 ± 0.36	1.44 ± 0.11	3.96 ± 0.47	21.75 ± 1.54
6 min/12 h	LPM	73 ± 0.08	1.63 ± 0.10	2.94 ± 0.32	70	3.09 ± 0.36	1.60 ± 0.13	7.19 ± 0.60	15.53 ± 1.62
	LPMSF	53 ± 0.09	1.25 ± 0.11	0.77 ± 0.43	36	1.27 ± 0.50	0.54 ± 0.27	4.36 ± 0.54	21.44 ± 1.62
**Statistical analysis**									
		*p*	*p*	*p*	*p*	*p*	*p*	*p*	*p*
Immersion time (IT)		0.001	0.023	0.001	0.001	0.001	0.001	0.001	0.038
Culture medium composition (CMC)		0.005	0.001	0.001	0.001	0.002	0.001	0.002	0.094
IT × CMC		0.507	0.567	0.67	0.824	0.164	0.046	0.002	0.057

Two-way analysis of variance (ANOVA), Tukey’s test (*p* < 0.05). LPM: liquid proliferation medium; LPMSF: liquid proliferation medium without sucrose. RGR: relative growth rate; SPAD: index of chlorophyll content in leaf.

**Table 4:** Effect of culture medium on the biochemical parameters of *Humulus lupulus* L., cv Columbus, plantlets grown in temporary immersion system (mean + standard error) after 4 weeks of culture.

Immersion Time	Culture Medium Composition	TPC (mg GAE/g)	AO (mg TEAC/g)
3 min/12 h	LPM	5.15 ± 0.13	5.55 ± 0.23
	LPMSF	5.05 ± 0.13	4.97 ± 0.23
6 min/12 h	LPM	4.94 ± 0.13	5.60 ± 0.23
	LPMSF	4.36 ± 0.19	2.31 ± 0.33
**Statistical analysis**			
		*p*	*p*
Immersion time (IT)		0.015	0.000
Culture medium composition (CMC)		0.054	0.000
IT × CMC		0.154	0.000

Two-way analysis of variance (ANOVA), Tukey’s test (*p* < 0.05). LPM: liquid proliferation medium; LPMSF: liquid proliferation medium without sucrose. TPC: total (poly)phenolic content; AO: antioxidant capacity.

## Data Availability

The original contributions presented in this study are included in the article; further inquiries can be directed to the corresponding authors.
